# Impacts of flooding on agricultural soil microbiome

**DOI:** 10.1128/spectrum.01108-26

**Published:** 2026-09-08

**Authors:** Eva Kilcoyne, Shauna O’Shea, Meghana Srinivas, Tatiana Garrido Gil, André Sousa, Ana Sofia Rodrigues dos Santos, Sophia Rosencrantz, Sophia Westphal, Stefanie Bär, Verena Jentzen, Juan Ignacio Vilchez, Ruben Rosencrantz, Fiona Walsh

**Affiliations:** 1 National University of Ireland 98865, Maynooth, Kildare, Ireland; 2 Kathleen Lonsdale Institute for Human Health Research, Maynooth, Kildare, Ireland; 3 Instituto de Tecnologia Química e Biologica (ITQB)-NOVA, iPlantMicro Lab, Oeiras, Lisboa, Portugal; 4 Fraunhofer Institute for Applied Polymer Research IAP, Potsdam, Germany; 5 Institute of Materials Chemistry, Brandenburg University of Technology Cottbus-Senftenberg, Senftenberg, Germany; Connecticut Agricultural Experiment Station, New Haven, Connecticut, USA

**Keywords:** soil microbiome, climate change, 16S rRNA amplicon sequencing, soil flooding

## Abstract

As a result of climate change, increased flooding events have been recorded across the globe. The role of soil microbiomes in soil health and fertility is well recognized, as these microbial communities drive essential processes such as nutrient cycling, organic matter decomposition, and plant health. The composition and function of these communities can be highly sensitive to environmental changes. The aim of this study is to identify the impacts of flooding on soil microbiome composition and diversity. Paired flooded and non-flooded agricultural soils were collected from Germany, Ireland, and Portugal, and characterized using full-length 16S rRNA gene and ITS amplicon sequencing. Following false-discovery-rate correction, flooding was not associated with significant differences in bacterial or fungal alpha diversity. In contrast, paired PERMANOVA detected small overall effects of flooding on bacterial Bray–Curtis, Jaccard, and unweighted UniFrac distances, and on fungal Bray–Curtis and Jaccard distances, although community composition was structured more strongly by country. At the taxonomic level, *Thermodesulfobacteriota* was more highly represented in flooded soils, whereas the fungal phyla *Ascomycota* and *Mortierellomycota* were less represented. Eight bacterial genus-level assignments differed between conditions after correction, whereas no fungal genus-level differences remained significant. Flooding was associated with some geographically dependent restructuring of bacterial and fungal communities rather than a consistent loss of microbial diversity. These findings demonstrate the importance of regional context when evaluating microbiome responses to flooding and identify the need for future studies that integrate microbial data with measured soil physicochemical characteristics.

**IMPORTANCE:**

Flooding is an increasingly important disturbance in agricultural soils, yet its effects on bacterial and fungal communities remain incompletely understood. By comparing matched flooded and non-flooded soils across Germany, Ireland, and Portugal, this study shows that flooding was associated with small shifts in community composition without a consistent reduction in alpha diversity. Geographic location explained the dominant patterns in community structure, while a limited number of bacterial and fungal taxa differed between flooding conditions. These findings indicate that microbiome responses to flooding are context-dependent and are expressed primarily as changes in community composition rather than uniform losses of richness or evenness. The study provides a cross-country baseline for future work linking flood-associated microbial changes to measured soil chemistry, flooding history, and agricultural recovery.

## INTRODUCTION

Agricultural soils harbor highly diverse microbial communities that are fundamental to ecosystem functioning, soil fertility, and sustainable crop production (1). Soil bacteria and fungi drive key biogeochemical processes including nutrient cycling, organic matter decomposition, and plant nutrient acquisition, while also contributing to soil structure and plant health (2). Consequently, changes in microbial community composition can have important implications for soil health and agricultural productivity. Soil microbial communities are shaped by a range of interacting environmental factors, including soil type, pH, land use, and climate, making them highly responsive to environmental disturbance (3).

Climate change is increasing the frequency and intensity of extreme precipitation events worldwide, resulting in more frequent flooding of agricultural land (4). Flooding alters the physical and chemical properties of soils by increasing water saturation, reducing oxygen availability, altering redox potential, and modifying nutrient availability (5–7). As water fills soil pores, oxygen diffusion is restricted, creating hypoxic or anoxic conditions and altering soil redox potential. Microorganisms with anaerobic or facultatively anaerobic metabolisms gain a competitive advantage, whereas taxa dependent on well-aerated soils decline (8–11). These environmental changes create selective pressures that act as environmental filters, reshaping ecological niches within the soil (12). Flooding may also alter microbial dispersal, resource availability, and species interactions, collectively driving community restructuring (13). Collectively, these processes restructure microbial communities, altering both their taxonomic composition and ecological functioning (10, 11, 14).

Although the effects of flooding on soil physicochemical properties are well established, its influence on soil microbial communities remains underexplored, particularly under natural agricultural flooding events. Previous studies have demonstrated that flooding can alter bacterial and fungal community composition; however, reported responses vary considerably among studies, with the magnitude and direction of change differing according to soil type, climate, flood duration, agricultural management, and geographical location (15–17). These inconsistencies suggest that microbial responses to flooding are highly context-dependent and may be strongly influenced by local environmental conditions (13, 15, 18). In addition to flooding, microbial communities are shaped by biogeographical processes, with climate and soil properties contributing to distinct regional community assemblies (19, 20). Consequently, it remains unclear whether flooding produces consistent patterns of microbial community restructuring across agricultural soils or whether geographical variation is the dominant driver of community assembly. Furthermore, because flooding simultaneously alters multiple soil physicochemical properties, distinguishing the direct effects of flooding from indirect effects mediated through changes in soil conditions remains a significant challenge.

High-throughput sequencing has transformed characterization of complex microbial communities by allowing direct, culture-independent analysis of nucleic acids from environmental samples (21). Amplicon sequencing of the bacterial 16S rRNA gene and fungal internal transcribed spacer (ITS) region provides a robust approach for investigating microbial diversity and community composition across clinical and environmental samples (22–24). These approaches have greatly improved our understanding of microbial ecology and provide an opportunity to investigate how large-scale environmental disturbances, such as flooding, influence bacterial and fungal communities across geographically distinct agricultural soils.

In this study, we characterized bacterial and fungal communities in paired flooded and non-flooded agricultural soils collected from Ireland, Germany, and Portugal using 16S rRNA gene and ITS amplicon sequencing. We investigated how flooding influences bacterial and fungal community diversity, composition, and taxonomic structure across three European agricultural regions, and assessed the relative contributions of flooding and geographical location to patterns of microbial community assembly. By comparing paired flooded and non-flooded agricultural soils across three European countries, this study provides new insights into how flooding restructures bacterial and fungal communities under natural field conditions and improves our understanding of the ecological consequences of climate-driven flooding for agricultural soils.

## MATERIALS AND METHODS

### Soil sampling

Agricultural soils were sampled from flooded and paired non-flooded sites in Ireland, Germany, and Portugal during 2022 and 2023. Flooded sites were selected from agricultural fields known locally to be susceptible to flooding and were inundated at the time of sampling following natural flooding events. The frequency and duration of previous flooding events were not quantified. Corresponding non-flooded sites were selected from the same site as each flooded location and had not experienced flooding during the same period, allowing comparisons while minimizing differences in local biogeochemical conditions.

At each sampling location, eight individual soil cores were collected to a depth of 10 cm using a stainless-steel soil corer. The eight cores were pooled to produce one composite soil sample. A total of 46 composite samples representing 23 flooded/non-flooded pairs were collected: 10 samples from Germany (5 pairs), 16 from Ireland (8 pairs), and 20 from Portugal (10 pairs).

Soil physicochemical characteristics were not measured as part of this study. Approximate soil classifications were instead assigned according to sampling location using publicly available national soil maps. Irish soil classifications were obtained from the General Soil Map of Ireland (1:575,000) and relevant county soil maps (1:127,560). Portuguese soils were classified using the Soil Map of Portugal (1:1,000,000) while German soil classifications were obtained from the Soil Map of the Federal Republic of Germany (1:1,000,000; WMS). These map-derived classifications were used only to provide descriptive site information and were not treated as measured physicochemical variables in the statistical analyses.

### DNA extraction and DNA quality control

Total genomic DNA was extracted from each composite soil sample using the DNeasy PowerSoil Pro Kit (Qiagen, Hilden, Germany) according to the manufacturer’s instructions. Four independent DNA extractions were performed for each soil sample. DNA integrity was assessed by electrophoresis on a 1.5% agarose gel prepared in 1 × TAE buffer to confirm the presence of high-molecular-weight genomic DNA. DNA purity was evaluated using a DeNovix DS-11 spectrophotometer (DeNovix Inc., Wilmington, DE, USA) by measuring the A260/A280 and A260/A230 absorbance ratios to assess potential protein and reagent contamination. DNA concentration was then quantified using a Qubit 1 Fluorometer with the Qubit dsDNA High Sensitivity Assay Kit (Thermo Fisher Scientific, Waltham, MA, USA) according to the manufacturer’s instructions. For each soil sample, the DNA extract with the highest DNA yield and intact genomic DNA was selected for downstream library preparation and sequencing.

### 16S rRNA and ITS amplicon sequencing

Full-length bacterial 16S rRNA gene and fungal internal transcribed spacer (ITS) amplicon sequencing were performed by Novogene (Cambridge, United Kingdom) using the PacBio Sequel II/IIe platform in HiFi circular consensus sequencing (CCS) mode. The bacterial full-length 16S rRNA gene (V1–V9) was amplified using primers 27F (5′-AGRGTTYGATYMTGGCTCAG-3′) and 1492R (5′-RGYTACCTTGTTACGACTT-3′), generating amplicons of approximately 1,500 bp (25). The fungal full-length ITS region (ITS1–5.8S–ITS2) was amplified using primers ITS9munngs (5′-TACACACCGCCCGTCG-3′) and ITS4ngsUni (5′-CCTSCSCTTANTDATATGC-3′), generating amplicons of approximately 500–800 bp (26).

PCR amplifications were performed in 15 μL reaction volumes containing Phusion High-Fidelity PCR Master Mix (New England Biolabs, USA), 0.2 μM of each primer, and approximately 10 ng of template DNA. Thermal cycling consisted of an initial denaturation at 98°C for 1 min, followed by 30 cycles of denaturation at 98°C for 10 s, annealing at 50°C for 30 s, and extension at 72°C for 30 s, with a final extension at 72°C for 5 min.

Each sequencing batch included internal positive and negative controls throughout PCR amplification, library preparation, and sequencing to monitor contamination and ensure sequencing quality. Amplicons were purified and converted into SMRTbell libraries according to the manufacturer’s protocol. Libraries were quantified using Qubit fluorometry, pooled, and sequenced on the PacBio Sequel II/IIe platform.

### Bioinformatic analysis

Raw PacBio HiFi reads were initially processed by Novogene using their amplicon-analysis workflow. Raw subreads were demultiplexed according to sample-specific barcode sequences using Lima, and circular consensus sequences were generated using SMRT Link v7.0 with a minimum of three passes and a minimum predicted accuracy of 0.99. Barcode and primer sequences were removed, and the sequences were quality filtered to generate high-quality clean reads. Chimeric sequences were identified and removed using UCHIME. Quality-control metrics, including the number of clean reads, total bases, and average read length for each sample, are provided in Table S2.

The bacterial 16S rRNA gene reads were analyzed using QIIME2 version 2026.1.0. Amplicon sequence variants were inferred using the DADA2 denoise-single plugin, with no additional trimming or truncation, a maximum expected-error threshold of 2, and consensus chimera removal. Representative bacterial sequences were aligned using MAFFT, and a rooted phylogenetic tree was constructed using FastTree. For the fungal data set, the ASV feature table and representative ITS sequences generated by Novogene were imported into QIIME2 version 2026.1.0 for downstream analysis.

Taxonomic classification was performed using the QIIME2 classify-sklearn method with a confidence threshold of 0.7. Bacterial ASVs were classified using the SILVA version 138.2 full-length classifier, while fungal ASVs were classified using the UNITE version 10 dynamic classifier. Features classified within Bacteria or Archaea were retained for bacterial taxonomic summaries, while features classified within the kingdom Fungi were retained for fungal analysis.

Alpha-rarefaction curves were used alongside sample-retention information to select appropriate sampling depths (Fig. S1). Sampling completeness was assessed using Good’s coverage. The bacterial feature table was rarefied to 600 sequences per sample, retaining 44 samples representing 22 complete flooded/non-flooded pairs. Samples I.Flooded3 and I.N.Flooded3 were excluded because they did not meet the selected depth. Bacterial alpha diversity was assessed using observed features, Shannon diversity, Pielou’s evenness, and Faith’s phylogenetic diversity. The fungi-only feature table was rarefied to 3,000 sequences per sample, and fungal alpha diversity was assessed using observed features, Shannon diversity, and Pielou’s evenness. Following rarefaction, 42 samples representing 21 complete flooded/non-flooded pairs were retained: 5 pairs from Germany, 8 from Ireland, and 8 from Portugal.

Statistical analyses were performed in R version 4.4.2. Data preparation was performed using the dplyr and tidyr packages. Alpha-diversity differences between flooded and non-flooded samples were tested using paired Wilcoxon signed-rank tests. Bacterial beta diversity was assessed using Bray–Curtis, Jaccard, unweighted UniFrac, and weighted UniFrac distances, while fungal beta diversity was assessed using Bray–Curtis and Jaccard distances. Community composition was visualized using principal coordinates analysis. Differences between paired flooded and non-flooded communities were tested using PERMANOVA with permutations restricted within sample pairs. Homogeneity of multivariate dispersion was assessed using PERMDISP. PERMANOVA and PERMDISP were performed using the vegan package, with restricted permutation schemes generated using the permute package. Analyses were conducted across all countries and separately within Germany, Ireland, and Portugal. *P* values were adjusted using the Benjamini–Hochberg false-discovery-rate procedure, with an adjusted *P* value below 0.05 considered statistically significant. Figures were generated using ggplot2, multipanel figures were assembled using patchwork, and genus-level heatmaps were generated using pheatmap.

Taxonomic-composition analyses were performed using the unrarefied, filtered feature tables. Phylum- and genus-level features were retained for formal testing when they occurred in at least 20% of samples and had a mean relative abundance of at least 0.1%. The prevalence threshold excluded highly sparse taxa detected in only a small number of samples, while the abundance threshold reduced testing of very-low-abundance taxa that may be sensitive to filtering and analytical choices (27). A pseudocount of 0.5 was added before centered log-ratio (CLR) transformation, and flooded and non-flooded samples were compared using paired Wilcoxon signed-rank tests. Benjamini–Hochberg correction was applied separately to the overall and country-specific sets of tests. Heatmaps show the 35 most abundant named genera; group means were log_10_-transformed after addition of 0.001 and standardized within each genus to row *Z*-scores.

## RESULTS

All soil samples were agricultural soils; however, approximate soil types were identified using sampling location. The main types of soils sampled in Portugal were fluvisols, cambisols, luvisols, and vertisols. The samples collected from various locations around Ireland comprised four main soil types: acid brown earth, humified fen peat, raised bog peat, and gray-brown podzolic. The German soil types sampled were found to be albeluvisols and histosols.

### Sequencing and feature-table characteristics

Taxonomic classification produced 11,445 bacterial 16S rRNA gene ASVs and 14,780 ITS ASVs. After taxonomic filtering, the feature tables contained 10,567 prokaryotic features and 6,959 fungal features across the 46 samples. Good’s coverage was 1.000 for all 44 bacterial samples retained for diversity analysis and for all 46 fungi-only ITS samples, indicating high sampling completeness (Tables S7 and S33). Per-sample sequencing and quality-control statistics are provided in Tables S2 and S6. Differences in within-sample richness were evaluated using the alpha-diversity analyses.

### Bacterial and fungal relative abundance

**Fig 1:**
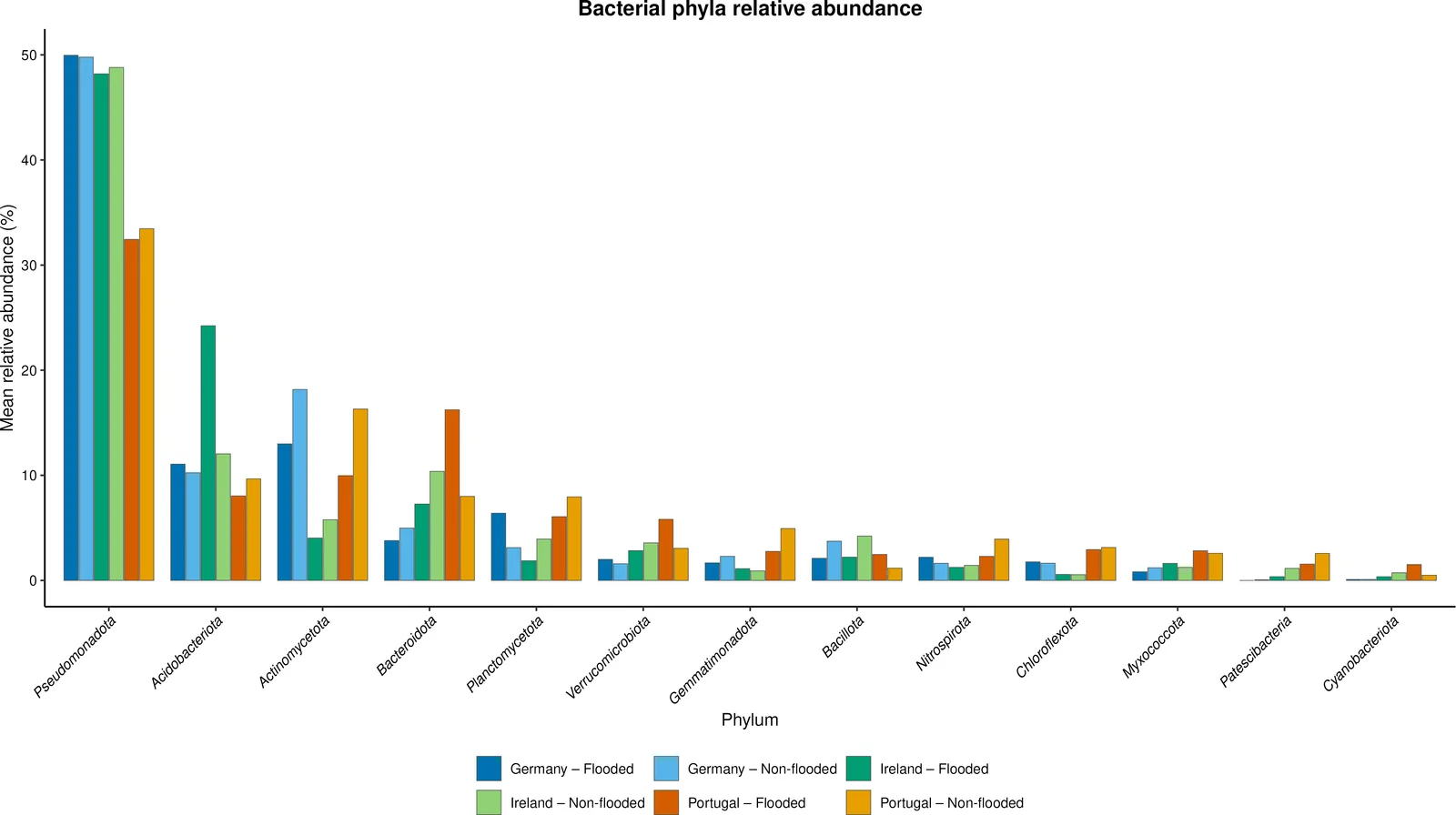
Relative abundance of the dominant bacterial phyla in flooded and non-flooded agricultural soils from Germany, Ireland, and Portugal. Bars show the mean relative abundance within each country and flooding group, calculated from the unrarefied prokaryote-only feature table. Groups comprised 5 flooded and 5 non-flooded samples from Germany, 7 of each from Ireland, and 10 of each from Portugal. Taxonomy was assigned using SILVA version 138.2.

The bacterial communities were dominated by *Pseudomonadota* (mean relative abundance, 41.74%), followed by *Acidobacteriota* (12.22%), *Actinomycetota* (11.07%), *Bacteroidota* (9.31%), and *Planctomycetota* (5.19%; Fig. 1). Other abundant phyla included *Verrucomicrobiota* (3.44%), *Gemmatimonadota* (2.52%), *Bacillota* (2.52%), *Nitrospirota* (2.28%), *Chloroflexota* (1.95%), *Myxococcota* (1.92%), and *Patescibacteria* (1.18%).

In paired CLR-based analysis of 22 phyla, *Thermodesulfobacteriota* was the only phylum that differed significantly between flooding conditions. It had a higher relative representation in flooded soils (median relative abundance, 0.426%) than in non-flooded soils (median, 0%; median CLR difference = 1.186; *P* = 0.000826; FDR-adjusted *P* = 0.0182). No phylum-level difference remained significant in the country-specific analyses (Table S17).

**Fig 2:**
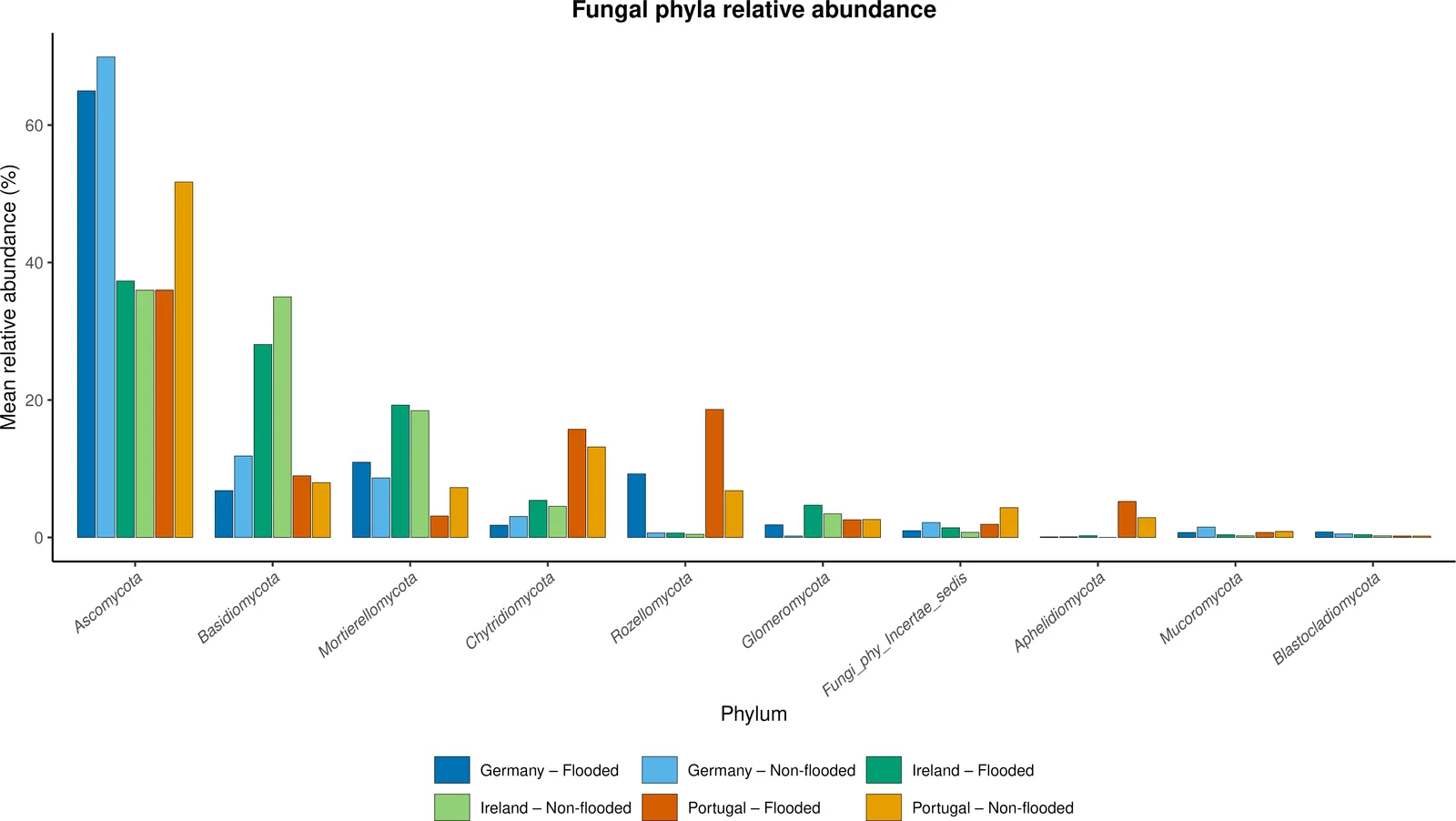
Relative abundance of the dominant fungal phyla in flooded and non-flooded agricultural soils from Germany, Ireland, and Portugal. Bars represent the mean relative abundance of each of the 10 most abundant classified fungal phyla within each country and flooding group, calculated from the unrarefied fungi-only feature table. Groups comprised 5 flooded and 5 non-flooded samples from Germany, 8 of each from Ireland, and 10 of each from Portugal. Taxonomy was assigned using the UNITE fungal reference database. Low-abundance and unclassified phyla are not displayed.

Fungal communities were dominated by *Ascomycota* (mean relative abundance, 46.47%), followed by *Basidiomycota* (16.68%), *Mortierellomycota* (10.95%), *Chytridiomycota* (8.54%), and *Rozellomycota* (6.80%). Together, these five phyla accounted for approximately 89.4% of the fungal community, although their relative representation varied among countries and flooding conditions (Fig. 2).

**Fig 3:**
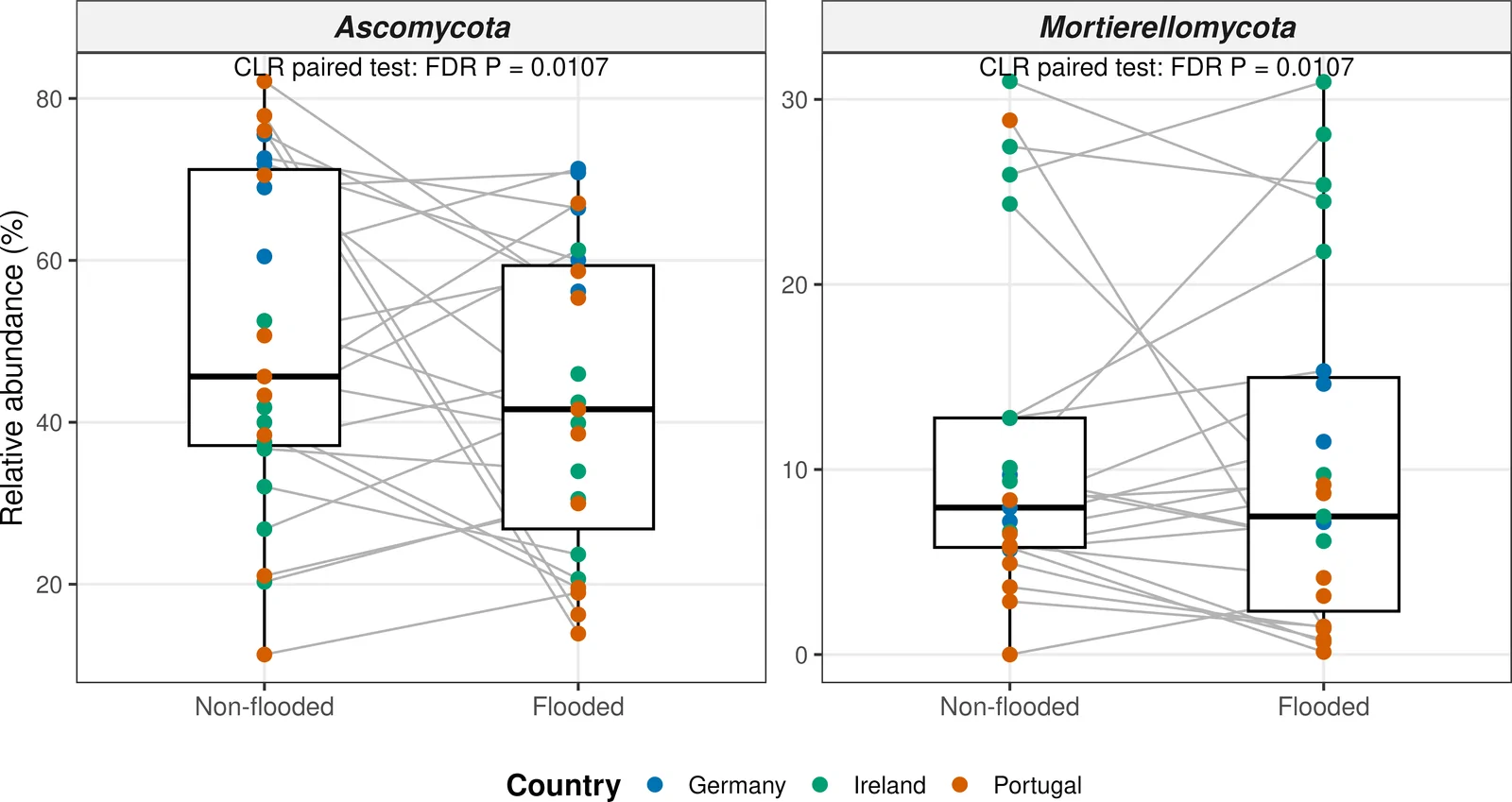
Relative abundance of fungal phyla that differed significantly between flooded and non-flooded agricultural soils. Paired comparisons are shown for Ascomycota and Mortierellomycota. Points represent individual samples and are colored according to country; gray lines connect the flooded and non-flooded members of each matched pair. Boxes show the median and interquartile range, with whiskers extending to 1.5 times the interquartile range. Statistical comparisons were performed using paired Wilcoxon signed-rank tests on centered log-ratio-transformed abundances across 23 matched pairs, with Benjamini–Hochberg false-discovery-rate correction across all tested phyla. The *y*-axes show the relative-abundance percentages.

Paired analysis of CLR-transformed abundances across the 23 matched pairs identified lower relative representation of *Ascomycota* in flooded soils (median relative abundance, 41.61%) than in non-flooded soils (45.66%; FDR-adjusted *P* = 0.0107). *Mortierellomycota* was also less highly represented in flooded soils (7.46%) than in non-flooded soils (7.94%; FDR-adjusted *P* = 0.0107; Fig. 3). No other tested phylum differed significantly following FDR correction, and no significant phylum-level differences were detected when Germany, Ireland, and Portugal were analyzed separately (Table S21).

### Bacterial alpha diversity

Following rarefaction to 600 sequences per sample, 44 samples representing 22 complete flooded/non-flooded pairs were retained for bacterial diversity analysis. Across all countries, the median Faith’s phylogenetic diversity was 23.9 in flooded soils and 25.5 in non-flooded soils, while the median numbers of observed features were 232 and 230, respectively. Median Shannon diversity was 7.25 in flooded soils and 7.37 in non-flooded soils, and median Pielou’s evenness was 0.928 and 0.949, respectively.

**Fig 4:**
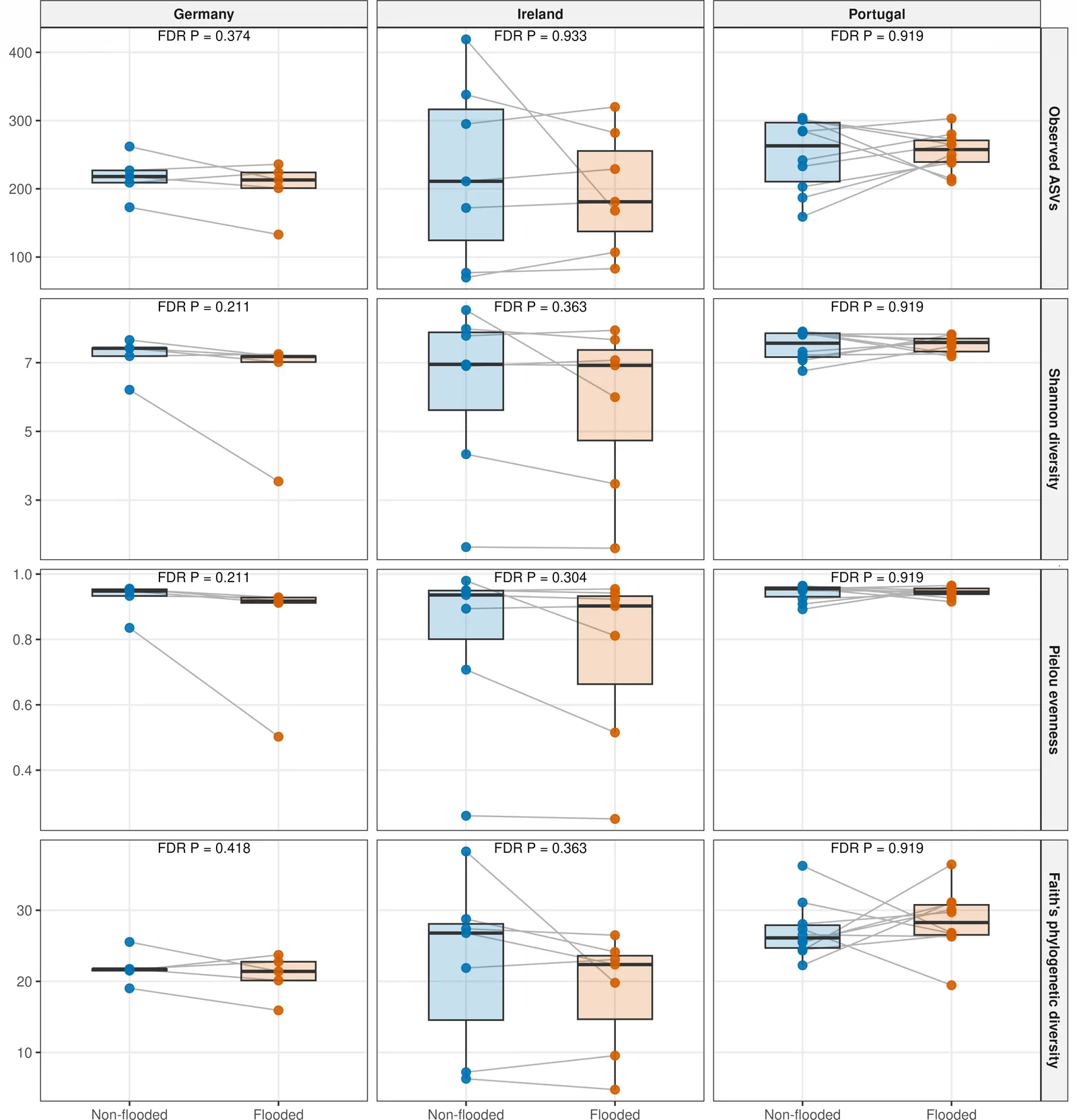
Bacterial alpha diversity in paired flooded and non-flooded agricultural soils from Germany, Ireland, and Portugal. Rows show observed ASVs, Shannon diversity, Pielou’s evenness, and Faith’s phylogenetic diversity. Blue and orange points represent non-flooded and flooded samples, respectively, with gray lines connecting matched pairs. Boxplots show the median and interquartile range. Differences were assessed using paired Wilcoxon signed-rank tests, with Benjamini–Hochberg FDR-adjusted *P* values shown above each panel. The feature table was rarefied to 600 sequences per sample.

Pielou’s evenness was lower in flooded soils before adjustment for multiple testing (paired Wilcoxon signed-rank test, *P* = 0.0377), but this difference was not significant following false-discovery-rate correction (adjusted *P* = 0.151). No significant overall differences were detected for Faith’s phylogenetic diversity, observed features, or Shannon diversity (adjusted *P* > 0.05). Similarly, no alpha-diversity metric differed significantly between flooded and non-flooded soils when Germany, Ireland, and Portugal were analyzed separately (adjusted *P* > 0.05; Fig. 4; Table S8).

### Fungal alpha diversity

Following rarefaction to 3,000 sequences per sample and exclusion of two incomplete Portuguese pairs, 42 samples representing 21 complete flooded/non-flooded pairs were retained for fungal diversity analysis. Across all countries, the median numbers of observed features were 242 in flooded soils and 249 in non-flooded soils. Median Shannon diversity was 6.25 in flooded soils and 6.34 in non-flooded soils, while median Pielou’s evenness was 0.810 and 0.803, respectively.

**Fig 5:**
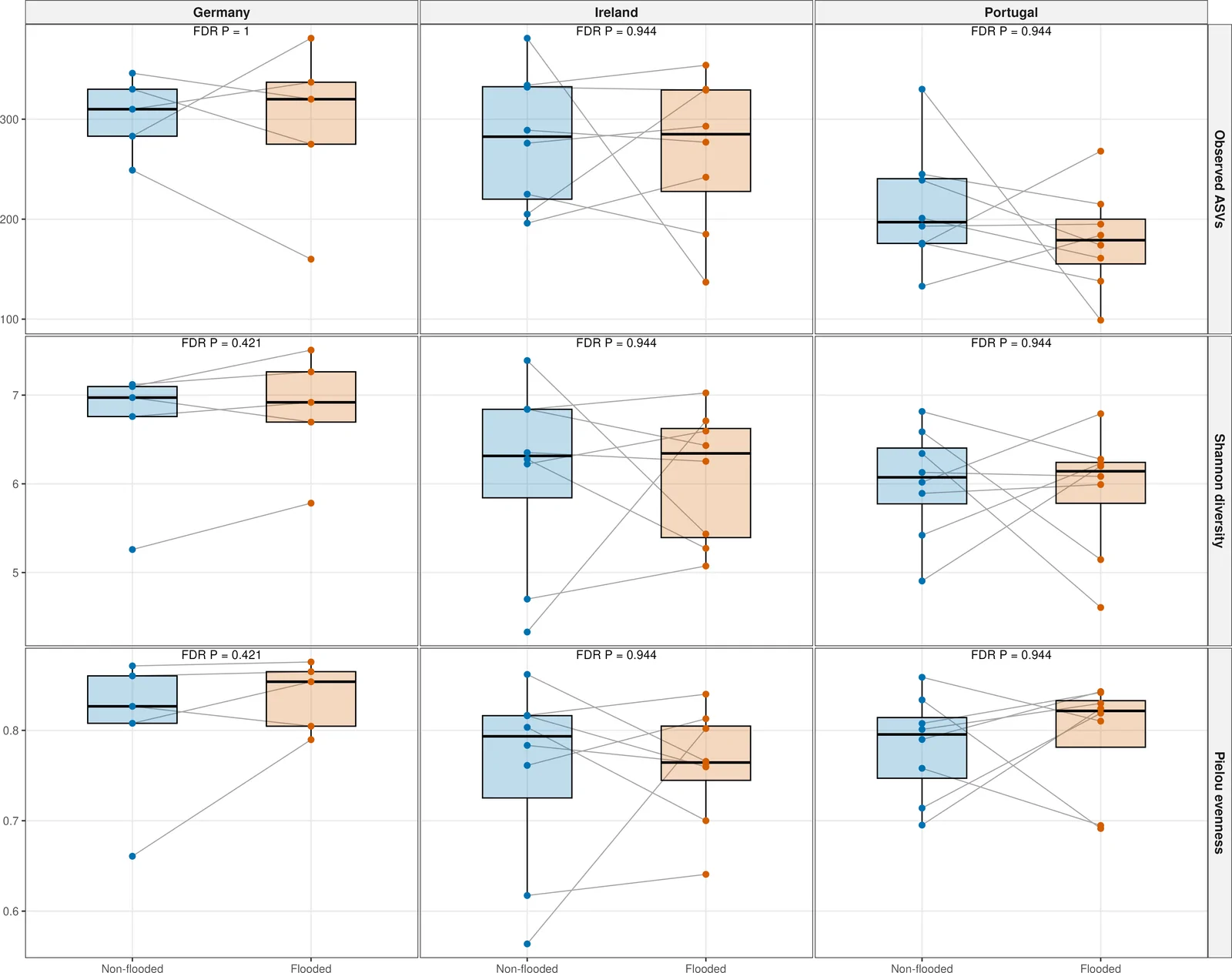
Fungal alpha diversity in paired flooded and non-flooded agricultural soils from Germany, Ireland, and Portugal. Rows show observed ASVs, Shannon diversity, and Pielou’s evenness. Blue and orange points represent non-flooded and flooded samples, respectively, with gray lines connecting matched pairs. Boxplots show the median and interquartile range. Differences were assessed using two-sided paired Wilcoxon signed-rank tests, with Benjamini–Hochberg FDR-adjusted *P* values shown above each panel. The fungi-only feature table was rarefied to 3,000 sequences per sample. Analyses included 21 complete pairs: 5 from Germany, 8 from Ireland, and 8 from Portugal.

No significant overall differences were detected for observed features, Shannon diversity, or Pielou’s evenness following false-discovery-rate correction (paired Wilcoxon signed-rank tests, adjusted *P* = 0.754 for all metrics). Similarly, no alpha-diversity metric differed significantly between flooded and non-flooded soils when Germany, Ireland, and Portugal were analyzed separately (adjusted *P* > 0.05; Fig. 5; Table S9).

### Bacterial beta diversity

**Fig 6:**
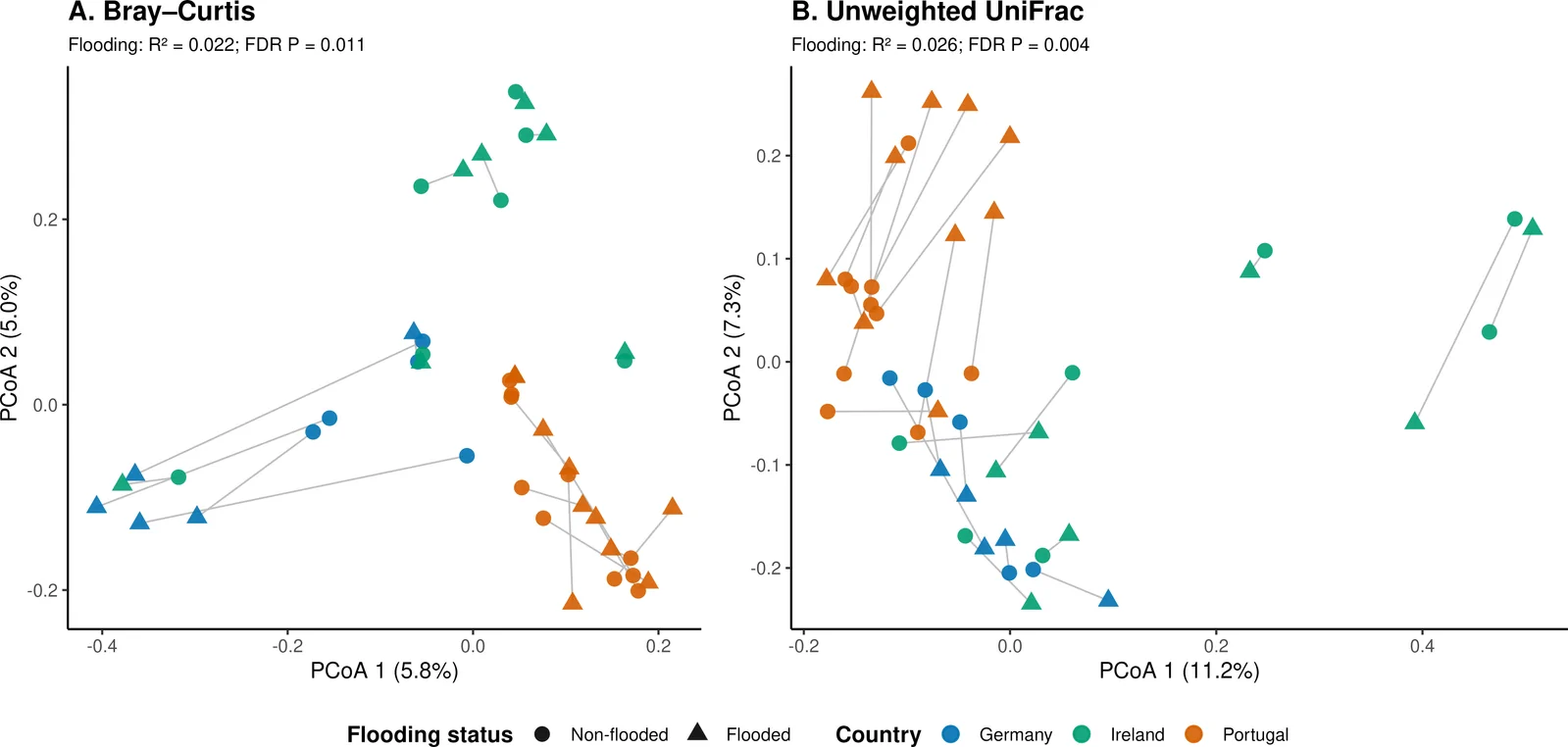
Principal coordinate analysis of bacterial communities in paired flooded and non-flooded soils based on (**A**) Bray–Curtis dissimilarity and (**B**) unweighted UniFrac distance. Colors represent countries, while circles and triangles represent non-flooded and flooded samples, respectively. Gray lines connect matched sample pairs. Axis labels show the percentage of variation explained. Flooding effects were assessed using PERMANOVA with 9,999 permutations restricted within pairs; *R*^2^ and Benjamini–Hochberg FDR-adjusted *P* values are shown above each panel. The feature table was rarefied to 600 sequences per sample.

Principal coordinates analysis indicated that bacterial communities were structured more strongly by country than by flooding status, with substantial overlap between flooded and non-flooded samples within countries (Fig. 6). However, paired PERMANOVA with permutations restricted within the 22 matched site pairs detected a small but significant overall effect of flooding on Bray–Curtis dissimilarity (*R*² = 0.0218, *P* = 0.0053, adjusted *P* = 0.0106), Jaccard distance (*R*² = 0.0226, *P* = 0.0089, adjusted *P* = 0.0119), and unweighted UniFrac distance (*R*² = 0.0263, *P* = 0.0010, adjusted *P* = 0.0040). Flooding was not associated with weighted UniFrac distance (*R*² = 0.0143, *P* = 0.5079, adjusted *P* = 0.5079).

When the countries were analyzed separately, Portugal was the only country in which flooding produced a significant difference following false-discovery-rate correction, based on unweighted UniFrac distance (*R*² = 0.0743, *P* = 0.00781, adjusted *P* = 0.0234). The apparent Portuguese Jaccard difference was not significant after correction (*P* = 0.0410, adjusted *P* = 0.0938). No significant country-specific differences were detected for the remaining distance metrics (adjusted *P* > 0.05). Tests for homogeneity of multivariate dispersion were nonsignificant within all countries, indicating that the country-specific PERMANOVA findings were not attributable to differences in within-group dispersion (Tables S10 to S12).

### Fungal beta diversity

**Fig 7:**
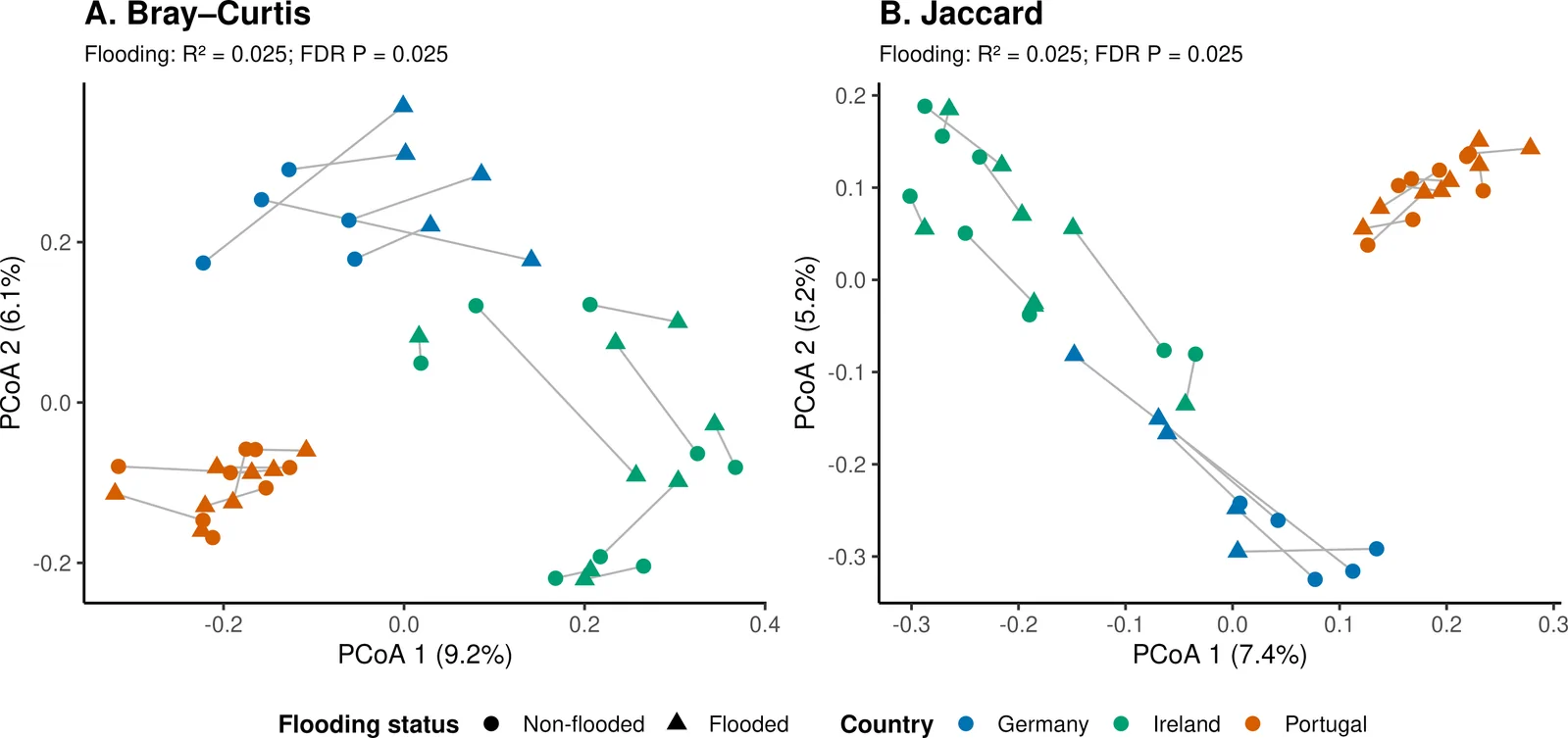
Principal coordinate analysis of fungal communities in paired flooded and non-flooded agricultural soils based on (**A**) Bray–Curtis dissimilarity and (**B**) Jaccard distance. Colors represent countries, while circles and triangles represent non-flooded and flooded samples, respectively. Gray lines connect matched sample pairs. Axis labels show the percentage of variation explained. Overall flooding effects were assessed using PERMANOVA with 9,999 permutations restricted within sample pairs; *R*^2^ and Benjamini–Hochberg FDR-adjusted *P* values are shown above each panel. The fungi-only feature table was rarefied to 3,000 sequences per sample, and analyses included 21 complete pairs.

Principal coordinate analysis indicated that fungal communities were structured more strongly by country than by flooding status, with substantial overlap between flooded and non-flooded samples within countries (Fig. 7). Paired PERMANOVA, with permutations restricted within the 21 matched site pairs, detected a small but significant overall effect of flooding on Bray–Curtis dissimilarity (*R*² = 0.0249, *P* = 0.0250, adjusted *P* = 0.0250) and Jaccard distance (*R*² = 0.0247, *P* = 0.0135, adjusted *P* = 0.0250). When the countries were analyzed separately, no significant differences between flooded and non-flooded soils were detected for either Bray–Curtis or Jaccard distances following false-discovery-rate correction (adjusted *P* > 0.05). Tests for homogeneity of multivariate dispersion were also nonsignificant both overall and within each country, indicating that the overall PERMANOVA findings were not attributable to differences in within-group dispersion (Tables S13 to S16).

### Bacterial and fungal genera associated with flooding and geographic location

**Fig 8:**
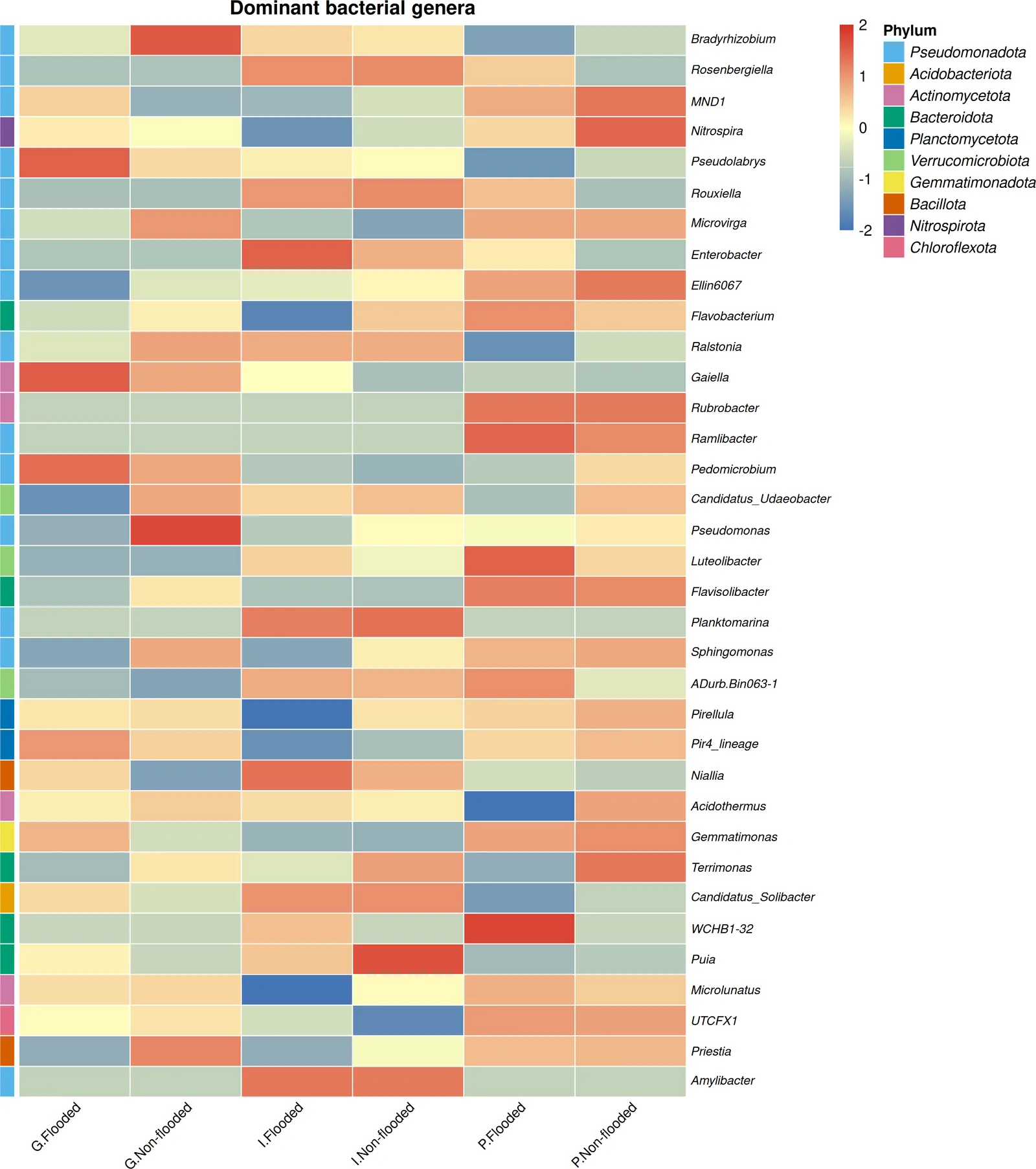
Heatmap showing the 35 most abundant named bacterial genera in Germany, Ireland, and Portugal. Values represent log-transformed, row-standardized mean relative abundances (*Z*-scores), with red indicating higher and blue indicating lower relative abundance within each genus. The row annotation denotes the phylum-level classification of each genus.

Based on genus-level annotations, the 35 most abundant named bacterial genera were visualized using a heatmap of mean relative abundance for each country and flooding condition (Fig. 8). In Germany, *Pseudolabrys*, *MND1*, and *Pedomicrobium* showed higher mean relative abundance in flooded soils, whereas *Bradyrhizobium*, *Pseudomonas*, and *Microvirga* showed higher mean relative abundance in non-flooded soils. In Ireland, *Enterobacter*, *Niallia,* and *Bradyrhizobium* showed higher mean relative abundance in flooded soils, whereas *Rouxiella*, *Rosenbergiella*, and *Flavobacterium* showed higher mean relative abundance in non-flooded soils. In Portugal, *Flavobacterium*, *Luteolibacter*, and *Ramlibacter* showed higher mean relative abundance in flooded soils, whereas *Nitrospira*, *Acidothermus*, and *Ellin6067* showed higher mean relative abundance in non-flooded soils.

Paired CLR-based analysis identified eight genus-level assignments that differed significantly between flooded and non-flooded soils across all 22 pairs. *Sphingomonas* showed lower relative representation in flooded soils (adjusted *P* = 0.0233), whereas *ADurb.Bin063-1*, *Sideroxydans*, *Ramlibacter*, *Anaerolinea*, *Luteolibacter*, *GOUTA6*, and *Woeseia* showed greater relative representation in flooded soils (adjusted *P* = 0.0233–0.0380). Several of these genera were sparsely distributed and had median relative abundances of zero; these results should therefore be interpreted cautiously. No genus-level differences remained significant when Germany, Ireland, and Portugal were analyzed separately (adjusted *P* > 0.05; Table S25). Consequently, the country-level patterns displayed in the heatmap should be interpreted as descriptive differences in mean relative abundance rather than statistically significant country-specific effects of flooding.

**Fig 9:**
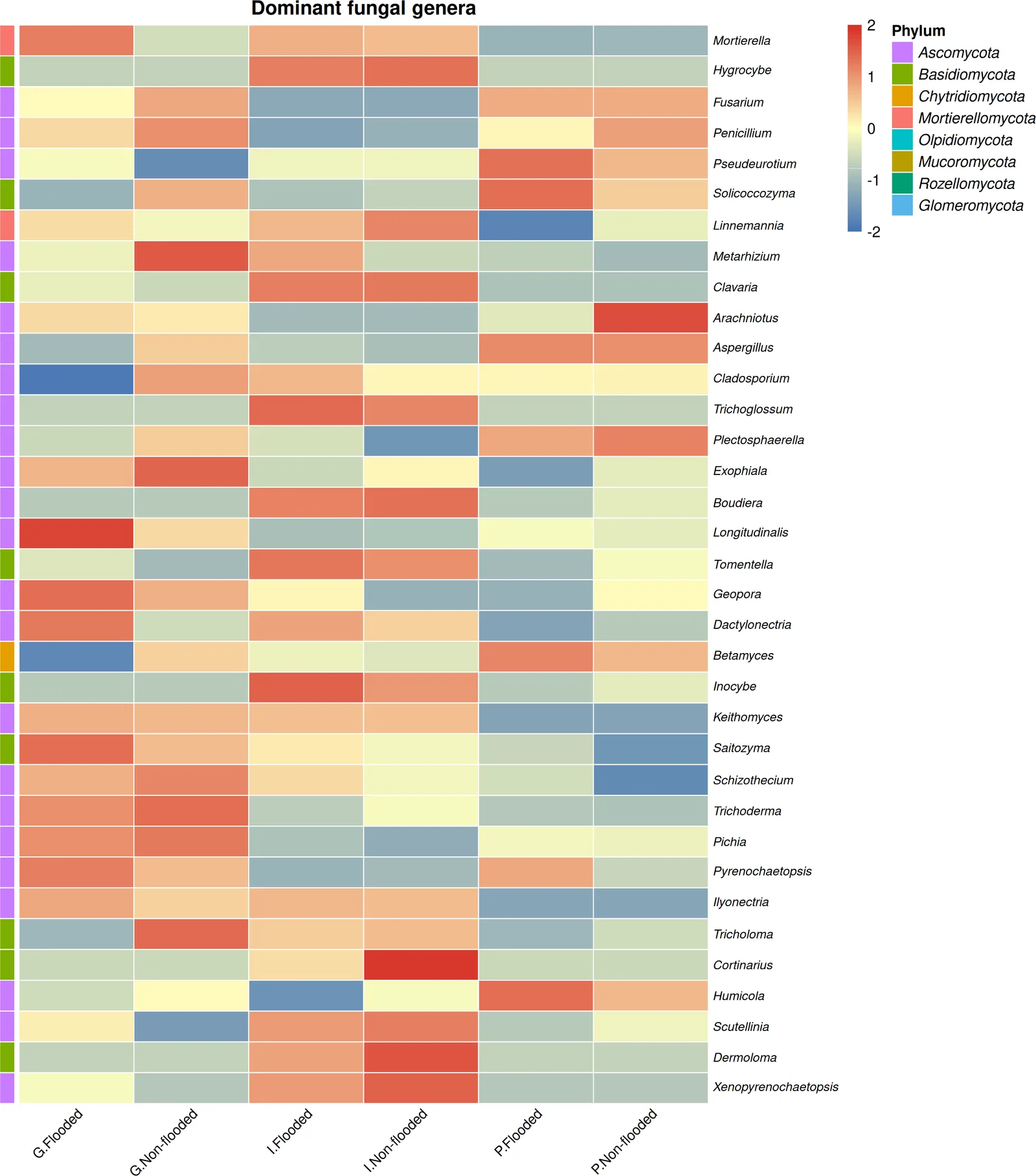
Relative abundance of dominant fungal genera in flooded and non-flooded soils. Heatmap showing the 35 most abundant fungal genera in Germany, Ireland, and Portugal. Values represent log-transformed, row-standardized mean relative abundances (*Z*-scores), with red indicating higher and blue indicating lower relative abundance within each genus. The row color denotes the phylum-level classification of each genus.

Based on genus-level annotations, the 35 most abundant named fungal genera were visualized using a heatmap of mean relative abundance for each country and flooding condition (Fig. 9). In Germany, *Mortierella*, *Geopora*, and *Longitudinalis* showed higher mean relative abundance in flooded soils, whereas *Exophiala*, *Metarhizium*, and *Cladosporium* showed higher mean relative abundance in non-flooded soils. In Ireland, *Hygrocybe*, *Clavaria*, and *Trichoglossum* were relatively abundant under both flooding conditions, while *Cortinarius* and *Dermoloma* showed higher mean relative abundance in non-flooded soils. In Portugal, *Pseudeurotium*, *Solicoccozyma*, and *Humicola* showed higher mean relative abundance in flooded soils, whereas *Arachniotus* and *Penicillium* showed higher mean relative abundance in non-flooded soils. Paired CLR-based analysis was performed on 74 fungal genera that met the prevalence and abundance thresholds. No genus differed significantly between flooded and non-flooded soils following false-discovery-rate correction, either across all 23 pairs or when Germany, Ireland, and Portugal were analyzed separately (adjusted *P* > 0.05; Table S29). The genus-level patterns displayed in the heatmap should therefore be interpreted as descriptive differences in mean relative abundance rather than statistically significant effects of flooding.

## DISCUSSION

The role of soil microbiomes in soil health and fertility is well recognized, as these microbial communities drive essential processes such as nutrient cycling, organic matter decomposition, and plant health (2, 28, 29). The composition and function of these communities can be highly sensitive to environmental changes. Flooding, which is becoming more frequent and intense, can drastically alter soil conditions including oxygen levels, nutrient availability, and pH (30). Flooding has also been shown to increase heavy metal pollution in soil (31). Understanding how flooding affects the soil microbiome is therefore important for predicting ecosystem responses to climate change and managing agricultural practices (32).

In this study, the effects of flooding on soil bacterial and fungal communities in Germany, Ireland, and Portugal were assessed by comparing paired flooded and non-flooded agricultural soils. No significant differences in bacterial or fungal alpha diversity were detected, either across all countries or when the countries were analyzed separately. This indicates that flooding did not cause a consistent reduction in microbial richness or evenness. However, small but significant differences in bacterial and fungal community composition were detected across all countries. The absence of changes in alpha diversity alongside changes in beta diversity suggests that flooding caused taxonomic turnover. Some microbial groups changed in their relative representation without causing an overall loss of diversity. Similar patterns, in which flooding or increased soil moisture altered community composition without causing a consistent reduction in alpha diversity, have been reported previously (33, 34).

Geographic location had a stronger influence on the microbial communities than flooding. The PCoA plots showed that bacterial and fungal communities clustered mainly according to country, while flooded and non-flooded samples generally overlapped within each country. Flooding explained approximately 1.4–2.6% of the overall variation in bacterial community composition and approximately 2.5% of the variation in fungal composition. Therefore, although the effects of flooding were statistically significant for several distance measures, they accounted for only a small proportion of the total variation. When the countries were analyzed separately, Portugal was the only country with a significant bacterial difference after correction for multiple testing, based on unweighted UniFrac distance. No country-specific fungal differences remained significant. These results suggest that the microbial response to flooding depends strongly on the community already present at each location. Strong effects of geographic location, soil type, and local environmental conditions on soil microbial community composition have also been reported (35, 36).

At the phylum level, *Pseudomonadota* was the most abundant bacterial phylum across the three countries and both flooding conditions. However, *Thermodesulfobacteriota* was the only bacterial phylum that differed significantly between flooded and non-flooded soils after correction for multiple testing and was more highly represented in flooded soils. Some members of this phylum are associated with anaerobic environments and sulfur cycling (37, 38), which may be relevant to the lower-oxygen conditions that can develop in flooded soils (39). However, oxygen levels and sulfur concentrations were not measured, so this possible explanation cannot be confirmed.

The fungal communities were dominated by *Ascomycota*, followed by *Basidiomycota*, *Mortierellomycota*, *Chytridiomycota*, and *Rozellomycota. Ascomycota* and *Mortierellomycota* had significantly lower relative representation in flooded soils. No other fungal phylum differed significantly, and no phylum-level differences were detected within the individual countries. This suggests that the fungal response to flooding was small and varied among locations. The lower representation of *Ascomycota* in flooded soils agrees with some previous studies (40, 41); however, other studies have reported an increase in *Ascomycota* in soybean-associated communities under flooding (42). These contrasting findings suggest that phylum-level responses may depend on soil, vegetation, and flooding conditions.

At the genus level, bacterial responses to flooding were not uniform across the three countries. Across all 22 pairs, *Sphingomonas* had lower relative representation in flooded soils, whereas *ADurb.Bin063-1*, *Sideroxydans*, *Ramlibacter*, *Anaerolinea*, *Luteolibacter*, *GOUTA6*, and *Woeseia* had higher relative representation. However, several of these assignments were not detected in many samples and had median relative abundances of zero, so these results should be interpreted cautiously. None of the country-specific comparisons remained significant after correction for multiple testing. However, the descriptive results showed that *Pseudomonas* and *Bradyrhizobium* had higher mean relative abundances in non-flooded German soils, while *Luteolibacter* had a higher mean relative abundance in flooded Portuguese soils. Some *Pseudomonas* strains have recognized plant-associated and biocontrol activities (43, 44), while some *Bradyrhizobium* strains can promote nodulation and nitrogen fixation (45). These functions cannot be assigned to the genera detected here because species and strains within the same genus may have different functions.

No fungal genus differed significantly between flooded and non-flooded soils after correction for multiple testing, either across all pairs or within the individual countries. The descriptive results showed that *Penicillium*, for example, had a higher mean relative abundance in non-flooded Portuguese soils. Although some *Penicillium* strains have been reported to benefit plant growth (46), the species and strains present in this study could not be determined. The bacterial and fungal heatmap patterns should therefore be interpreted as variation among countries and sites rather than evidence that flooding consistently changed particular microbial functions.

This study examined microbial communities under natural flooding conditions across geographically distinct agricultural soils. The paired sampling design allowed flooded and non-flooded soils from comparable locations to be assessed, but soil chemical and physical properties were not measured. Flooding can alter oxygen availability, redox potential, pH, nutrient concentrations, and other soil properties, which may in turn affect microbial communities.

Overall, flooding was associated with small changes in bacterial and fungal community composition but did not cause a consistent reduction in microbial alpha diversity. The stronger separation of communities by country demonstrates that geographic location and local environmental conditions were more important in structuring the soil microbiome than flooding alone. The significant beta-diversity results indicate taxonomic turnover, with changes in community membership or relative representation occurring without an overall loss of diversity. Future studies should include measurements of soil chemical and physical properties, flooding duration, and microbial absolute abundance. Combining these measurements with metagenomic or transcriptomic analysis would help determine the mechanisms and functional consequences of microbial community changes in flooded agricultural soils.

## Data Availability

All sequencing data generated in this study are available in the European Nucleotide Archive (ENA), hosted by EMBL-EBI, under study accession number PRJEB85669. Individual sequencing accession numbers are provided in Table S1. Statistical results, quality-control information, and figure source data are provided in the Supplemental material. Bioinformatic processing was conducted using QIIME 2, which is publicly available at https://qiime2.org. Statistical analyses and figure generation were performed in R using the packages described in the Materials and Methods.

## SUPPLEMENTAL MATERIAL

Supplemental figure legend spectrum.01108-26-s0001.docx Legend for Fig. S1.

Figure S1 spectrum.01108-26-s0002.tiff Rarefaction curves for bacterial and fungal communities in agricultural soils.

Tables S1 to S33 spectrum.01108-26-s0003.xlsx Metadata, quality-control information, diversity statistics, taxonomic analyses, and figure source data.

ASM does not own the copyrights to Supplemental Material that may be linked to, or accessed through, an article. The authors have granted ASM a non-exclusive, world-wide license to publish the Supplemental Material files. Please contact the corresponding author directly for reuse.
