# Supplementary material for: Impacts of flooding on agricultural soil microbiome
Source: Microbiol Spectr. 2026 Sep 8;14(10):e01108-26. doi: 10.1128/spectrum.01108-26 (PMC13688923; doi:10.1128/spectrum.01108-26)
Supplement: Supplementary file 1 [file spectrum.01108-26-s0001.docx]

**Supplemental Figure S1**. Rarefaction curves for bacterial and fungal communities in agricultural soils. Rarefaction curves show the number of observed amplicon sequence variants (ASVs) as a function of sequencing depth for (A) 16S rRNA gene bacterial communities and (B) internal transcribed spacer (ITS) fungal communities. Each line represents an individual flooded or non-flooded soil sample and is coloured by country of origin: Germany (blue), Ireland (green) and Portugal (orange). Dashed vertical lines indicate the selected rarefaction depths used for downstream diversity analyses: 600 sequences per sample for bacterial communities and 3,000 sequences per sample for fungal communities.
